# The epidemiological background of small ruminant lentivirus infection in goats from Romania

**DOI:** 10.14202/vetworld.2020.1344-1350

**Published:** 2020-07-15

**Authors:** Adrian-Valentin Potarniche, Constantin Gheorghe Cerbu, Michal Czopowicz, Olga Szalus-Jordanow, Jaroslaw Kaba, Marina Spinu

**Affiliations:** 1 Department of Infectious Diseases and Preventive Medicine, Faculty of Veterinary Medicine, University of Agricultural Sciences and Veterinary Medicine Cluj-Napoca, Cluj, Romania; 2 Division of Veterinary Epidemiology and Economics, Institute of Veterinary Medicine, Warsaw University of Life Sciences, Warsaw, Poland; 3 Department of Small Animal Diseases with Clinic, Institute of Veterinary Medicine, Warsaw University of Life Sciences, Warsaw, Poland

**Keywords:** epidemiology, goat, outbreaks, small ruminant lentivirus

## Abstract

**Background and Aim:**

Caprine arthritis-encephalitis (CAE) is an economically significant viral disease of goats caused by a small ruminant lentivirus (SRLV) belonging to *Retroviridae* family. This study aimed to summarize current information on the epidemiological status of SRLVs infection in the population of goats from Romania and to point out the CAE incidence throughout the 2008-2018 periods.

**Materials and Methods:**

An exhaustive review of the papers published in the international literature concerning the epidemiological status of CAE in Romania was carried out using electronic databases, and available statistical data from the World Organization for Animal Health (OIE) regarding the incidence of the disease between 2008 and 2018 were analyzed.

**Results:**

The true individual-level seroprevalence of CAE was estimated in 13 of 42 counties (31%) and ranged from 0.4% to roughly 40%. One hundred eighty-two outbreaks from 14 counties (33%) were reported, with a peak in 2010.

**Conclusion:**

The findings sourcing in the literature are very scarce and show disagreement with the situation reported by the national veterinary authorities. Lack of SRLVs screening policies represents the main obstacle in limiting the spread of the disease. Romania’s National Sanitary Veterinary and Food Safety Authority should implement a program for diagnosis and surveillance of the disease to build a straightforward epidemiological picture that represents a prerequisite of any control and eradication program.

## Introduction

Caprine arthritis-encephalitis (CAE) is one of the most important viral diseases of goats, which causes significant economic losses all around the world [1]. CAE was first described by Cork *et al*. [2] in 1974, and the causal agent was first isolated from an arthritic goat by Crawford *et al*. [3] a few years later. CAE is produced by a single-stranded RNA lentivirus belonging to the family *Retroviridae*, of the *Ortervirales* order [4]. CAE virus (CAEV) and Maedi-Visna virus (MVV), both belonging to the genus *Lentivirus*, are grouped in small ruminant lentiviruses (SRLVs) due to their genomic and antigenic similarities [5]. SRLVs transmission mainly occurs by the ingestion of virus-containing colostrum/milk by the kids, but direct contact with the infected animals (horizontal transmission) should also be considered [6-8]. The disease is characterized by a long incubation period and persistent infection. In general, infected animals do not show clinical signs. In animals clinically ill, arthritis, mastitis, and pneumonia are the most frequent signs in adult goats, while kids show neurological signs [9]. The disease spreads subtly in a herd, affecting a high percentage of goats before the first clinical signs can be noticed [10]. Therefore, CAE diagnosis relies on laboratory tests. Even though detecting SRLVs by the use of molecular biology methods (polymerase chain reaction) appear to be a sensitive diagnostic approach, no such tests have been so far introduced in routine practice, and serological assays, such as enzyme-linked immunosorbent assay (ELISA) or agar-gel immunodiffusion (AGID), remain the main tests for CAE diagnosis [11]. Live trade of goats from countries where the disease has been reported is believed to be the main reason for its widespread [6]. At present, there are no treatments or vaccines against CAE. Thus, control programs remain the only way solution to avoid the spreading of SRLVs infection [12].

Little information is available regarding SRLVs infection in goats from Romania. CAE is a transmissible disease of goats which is subjected to the internal notification stipulated in Annex no.1 of the Order of the President of Romania’s National Sanitary Veterinary and Food Safety Authority (NSVFSA) no.79/2008. However, no surveillance or diagnosis programs are active at the national level. This is the first study that comes to put together and discusses all the information available on the SRLVs infection in goats from Romania.

The aim of this study was to review the actual epidemiological situation of SRLVs infection in goats in Romania based on scientific literature and to analyze the CAE incidence throughout the 2008-2018 period.

## Materials and Methods

### Ethical approval

No ethical approval was needed to perform this study.

### Searching approach

All the materials published in the international literature concerning the epidemiological status of CAE in Romania were reviewed using search engines such as Web of Science, PubMed, Scopus, ScienceDirect, and Google Scholar. The following keywords were used (alone or in combinations) for this purpose: “CAE,” “CAEV,” “SRLV,” “epidemiology,” “prevalence,” “seroprevalence,” “incidence,” “outbreaks,” “reports,” “detection,” “goats,” and “Romania.” Likewise, the available information from the World Animal Health Information Database (OIE), Food and Agriculture Organization (FAO) of United Nations, and from Romania’s NSVFSA was used.

### Data extraction

Data concerning goat characteristics (age, gender, breed, and herds size), samples (size, and specimen), counties investigated (name, and number), diagnostic methods used, and the prevalence (overall, individual-level, and herd-level) were extracted from the studies. Similarly, information about the size of the goat population, number of CAE outbreaks by years, and counties from Romania were collected from OIE, FAO, and NSVFSA databases.

### Statistical analysis

Sensitivity (Se) and specificity (Sp) of diagnostic tests used for the detection of SRLVs infection were based on so far published studies. When compared to the western-blotting, Se and Sp of AGID are roughly 76% and 98%, respectively [13], and its lower than for the whole-virus ELISA, which has Se and Sp of roughly 98% [14]. On the basis of these figures, the true seroprevalence was calculated using the Rogan-Gladen equation [15]. The 95% confidence intervals (CI) were calculated with the Wilson score method [16].

## Results

Only five studies regarding the occurrence of SRLV infection in goats in Romania have been published [17-21]. All of them were serological surveys, and three were carried out with the use of AGID test [18-20], and two with the use of the whole-virus ELISA [17,21].

**Figure-1:**
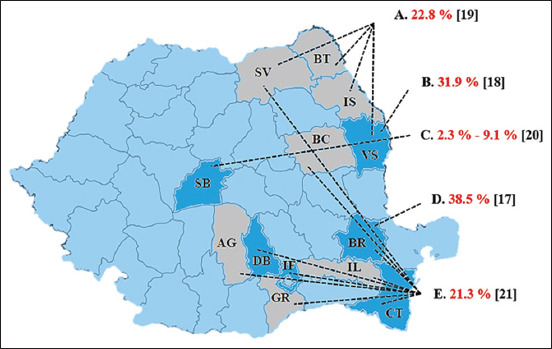
Map of the surveyed Romanian counties and the apparent seroprevalence of caprine arthritis-encephalitis. Counties: AG=Arges, BC=Bacau, BR=Braila, BT=Botosani, CT=Constanța, DB=Dimbovita, GR=Giurgiu, IF=Ilfov, IL=Ialomita, IS=Iasi, SB=Sibiu, SV=Suceava, VS=Vaslui.

Gurau *et al*. [17] tested 78 goats from a single herd counting 120 goats, located in the Braila County using an indirect ELISA kit (IDEXX CAEV/MVV Total Ab Test, Switzerland). Thirty tested positive, which yielded a true individual-level seroprevalence of 38.0% (CI 95%: 28.0%, 49.1%). Similarly, Mihai *et al*. [18] screened a 400-French Alpine goat herd from Vaslui County with AGID (CAEV-p28 AGID diagnostic kit, Pourquier-Montpellier, France). Ninety-four of 295 serum samples tested were positive, and the true individual-level seroprevalence was 40.4% (CI 95%: 34.9%, 46.0%). In the second study, the same authors screened a group of 412 symptomatic and asymptomatic goats from four Romanian counties. Of them, 94 tested positive in AGID test, resulting in a true individual-level seroprevalence of 28.1% (24.0%, and 32.7%) [19]. In a 3-year survey study in Sibiu county, Potarniche *et al*. [20] tested 15 947 sera with AGID (Pourquier-Montpellier, France). An overall true individual-level seroprevalence ranged from 0.4% (CI 95%: 0.3%, 0.6%) in the 1^st^ year to 9.1% (CI 95%: 8.5%, 10.7%) in the last year. Another study was conducted by Enache *et al*. [21], which tested 47 pooled samples (from 235 goats) originating from nine Romanian counties using a commercial ELISA kit (IDEXX CAEV/MVV Total Ab Test, Switzerland). The pooled sera were created by mixing 100 μl of serum from five animals from the same herd. Positive results were obtained for pooled samples from four counties (Dambovita, Ilfov, Braila, and Constanta). The results were variable from region to region with an overall seroprevalence of 21.3%. In none of these larger studies [19-21], the number of goat herds tested was mentioned. In total, 13 counties had been investigated, and the presence of the disease was reported in six of them (46%) (Figure-1 and Table-1) [17-21].

**Table -1:** Baseline features of comprised studies.

ID	No. of counties investig-ated	Period	Diag.	No. herds	Goats		Sample size	Pos. (+) samples		Apparent prevalence (CI 95%) (%)	True prevalence (CI 95%) (%)	Ref.
	
					No.	Signalment		No.	Additional information			
1	1	-	ELISA	1	120	-	78	30	28 ♀ (< 2 years old) 2 ♂ (<4 years old)	38.5 (28.4, 49.6)	38.0 (28.0, 49.1)	[17]
2	1	2014-2016	AGID	1	400	Breed: French Alpine Age: 1.5 – 4 years Gender: botd	295	94	89 ♀ (< 3 years old) 5 ♂ (< 3 years old)	31.9 (26.8, 37.4)	40.4 (34.9, 46.0)	[18]
3	4	2014-2017	AGID	-	8628	Breed: Carpatdian White Banat, Saanen, French Alpine, Murcian Age: all ages Gender: botd	412	94	< 3 years old	22.8 (19.0, 27.1)	28.1 (24.0, 32.7)	[19]
4	1	2009 (1^st^ year)	AGID		-		5621	129	-	2.3 (1.9, 2.7)	0.4 (0.3, 0.6)	[20]
		2010 (2^nd^ year)			-		7766	275	-	3.5 (3.2, 4.0)	2.1 (1.8, 2.4)
		2011 (3^rd^ year)			-		2560	232	-	9.1 (8.0, 10.2)	9.1 (8.5, 10.7)
5	9	-	ELISA		-		47 pooled sera (from 235 goats)	10	-	21.3 (12.0, 34.9)	Cannot be calculated for pooled sera as Se and Sp of ELISA performed on pooled samples is unknown	[21]

**Figure-2:**
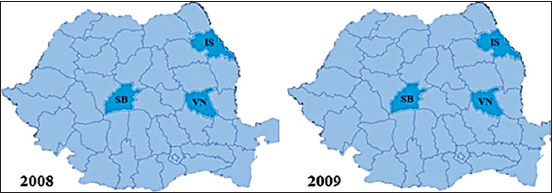
Distribution of the reported caprine arthritis-encephalitis outbreaks in Romania between 2008 and 2009. Counties: IS=Iasi, SB=Sibiu, VN=Vrancea.

**Figure-3:**
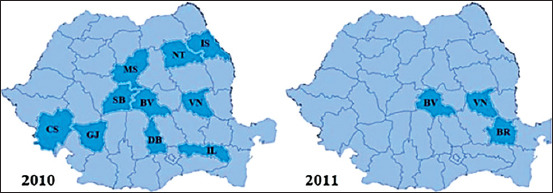
Distribution of the reported caprine arthritis-encephalitis outbreaks in Romania between 2010 and 2011. Counties: BR=Braila, BV=Brasov, CS=Caras-severin, DB=Dimbovita, GJ=Gorj, IL=Ilfov, IS=Iasi, M=Mures, NT=Neamt, SB=Sibiu, VN=Vrancea.

**Figure-4:**
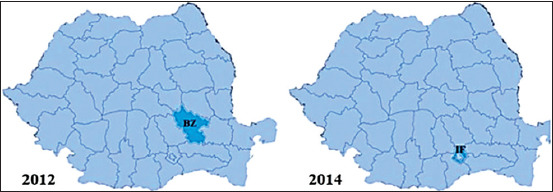
Distribution of the reported caprine arthritis-encephalitis outbreaks in Romania between 2012 and 2014. Counties: BZ=Buzau, IF=Ilfov.

**Figure-5:**
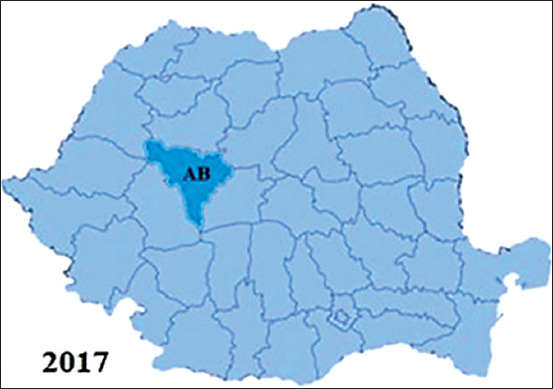
Distribution of the reported caprine arthritis-encephalitis outbreaks in Romania during 2017. Counties: AB=Alba.

The number of official outbreaks of CAE from Romania was quantified based on the data from OIE [22], which basically represents the result of serological investigations carried out by territorial structures of NSVFSA [23] after clinical cases of CAE have been suspected and reported by local veterinarians. The results regarding the incidence of CAE outbreaks in Romania according to the OIE are presented briefly in Figures-2-5. The first outbreak was noticed in 2008, in Sibiu County. Then, six more outbreaks were reported in the same county, with 49 cases of CAE confirmed using AGID. During the subsequent years, more and more outbreaks emerged in the whole country. The highest number of disease outbreaks was recorded in 2010 when a number of ten counties were involved. The last outbreak was reported at the beginning of the year 2017 in Alba-Iulia, where four goats were found to be positive after serological investigation (AGID). During 2013, 2015, 2016, and 2018, no CAE outbreaks were reported. Thus, throughout the 2008-2018 period, the disease was reported in 14 counties (33%) from Romania that yields 182 outbreaks (Table-2).

**Table-2:** The incidence of CAE outbreaks in Romanian counties throughout 2008-2018 period.

Location (counties)	Years											Total

	2008	2009	2010	2011	2012	2013	2014	2015	2016	2017	2018	
Alba										1		1
Braila				1								1
Brasov			3	1								4
Buzau					1							1
Caras-Severin			7									7
Dimbovita			9									9
Gorj			11									11
Ialomita			13									13
Iasi	1	5	2									8
Mures			1									1
Neamt			64									64
Sibiu	7	1	14									22
Vrancea	15	6	16	2								39
Ilfov							1					1
Total (14)	23	12	140	4	1		1			1		182

## Discussion

Under these circumstances, it is difficult to define the real epidemiological status of CAE in Romania given the low number of counties investigated (31%). Furthermore, the number of herds and individual goats that were taken into account was low in certain counties, only one herd being investigated. The overall seroprevalence of SRLVs infection in goats has been reported in several countries. Similar seroprevalence (2.9-38.5%) that the ones reported in Romania were found in India (3.3%) [24], Oman (5.1%) [25], Sudan (5.8%) [26], Belgium (6%) [27], Malaysia (8.9%) [28], Japan (10%) [29], Liban (13.1%) [30], Kosovo (15.6%) [31], Italy (18.6%) [32], and Algeria (29.7%) [33]. Lower seroprevalence was reported in Switzerland (0.06%) [34] and higher in Brazil (49.5%) [35], Poland (51.6%) [36], Croatia (53.7%) [37], and Taiwan (61.7%) [38].

According to Enache *et al*. [39] between 2006 and 2015, Romania had the highest number of CAE serological positive results reported in the whole Europe. In this regard, there are numerous factors that can influence the emergence of new outbreaks.

First of all, the low number of SRLVs outbreaks in some European countries was correlated with the existence of control and eradication programs. Moreover, management practices affect the prevalence of SRLVs in a herd. Therefore, control programs have been implemented in many countries since SRLVs were detected in their goat herds [40]. For example, Switzerland began a voluntary SRLVs eradication program in 1984, and in 1998 was recognized as a “CAE-free” country [6]. Similarly, Norway initiated programs aiming to eradicate the SRLVs infection [41,42]. Although CAE represents a disease subject to internal notification, no surveillance or diagnosis programs are currently implemented in Romania. Singularly, an active surveillance program was applied in 2010 due to the Order of the President of NSVFSA no.2/2010, which can explain the large number of outbreaks found during that year. According to it, 2% of goats from each herd were randomly tested. In Romania, both serological tests, AGID and ELISA are used as confirmatory methods by accredited laboratories [23]. Concisely, the following practices are recommended to be applied in SRLVs infected goat herds: (a) Permanent isolation of kids immediately after birth; (b) feeding of heat-treated colostrum (45°C for 60 min); (c) serological examination of the herd (twice per year) and maintaining the seronegative and seropositive goats separately; and (4) culling of seropositive goats [8,40].

The size of the goat population and the live animal trade represents other possible factors affecting the epidemiology of CAE. The goat population from Romania has increased every year, reaching 1.5 million goats in 2017. It is one of the biggest population of goats from Europe after Russia (2 mils), Spain (3.1 mils), and Greece (6.3 mils) [43]. In Romania, the imports of live sheep and goats from the intra-community area have increased in the period 2010-2014 by 9.3 times [44]. In recent years, imports of goats from breeds specialized in milk production, such as Saanen and French Alpine have increased in Romania. The animals were brought from different countries such as France or even New Zeeland, which are not free of SRLVs infection [19]. By comparison, looking at a Polish herd where the prevalence has increased from 15% to 75%, after the importation of dairy goats from France during the 1990s [6,8], we can presume the importation of goats to Romania as one of the causes of the latter increase in CAE prevalence. The virus is hardly found in native breeds unless they were exposed or had direct contact with imported goats [24]. Thus, the regulations regarding the live trade of goats stipulated in the national strategic program (Order of the President of NSVFSA no.35/2016) should be implemented very strictly to minimize the importation of infected animals.

Another critical aspect of the spreading of the virus is the cohabitation of sheep with goats. In Romania, farmers mostly keep both species together in the same shed or in close contact. Commonly, during the summer, sheep, and goats share grazing areas and water sources. This can increase the risk of SRLVs interspecies transmission. Phylogenetic analysis of SRLVs revealed that different SRLVs strains could be transmitted naturally and experimentally from sheep to goats and vice-versa [45,46]. Consequently, mixed farms of goats and sheep may represent an active source for the evolution of these viruses [47]. Therefore, the eradication programs implemented in both species could help in controlling the disease [48]. Moreover, recent studies have identified SRLVs in wild ruminants, which may also contribute to SRLVs epidemiology [49]. Likewise, the slow natural spread of the virus, the absence of clinical signs, or their misinterpretation by veterinarians can lead to unreported outbreaks.

## Conclusion

The available information regarding the situation of CAE in Romania is very scarce. There are only a few studies about the prevalence of CAE, therefore offering an incomplete picture of the actual status of the disease. The presence of the disease in the country is also confirmed by the last reported outbreaks. Lack of SRLVs screening policies represents the main obstacle in limiting the spread of the disease. However, SRLVs infection often shows a complex scenario. The infection is frequently subclinical, SRLVs are genetically related, and interspecies transmission is possible. Therefore, knowing the epidemiological dynamics, the need for surveillance of SRLVs infection remains a longstanding task. Romanian national veterinary authorities should implement a program for diagnosis and surveillance to build a straightforward epidemiological picture that represents a precondition of any control and eradication program.

## Authors’ Contributions

AVP designed the study, contributed to literature collection, prepared the figures/tables, and wrote the manuscript draft alongside with CGC. MC performed the statistical and epidemiological analyses. OS and MS critically revised the manuscript. JK provided conceptual support and critically reviewed the manuscript. All authors read, revised, and approved the final manuscript.
